# Alteration of the HIF-1α/VEGF Signaling Pathway and Disruption of the Cell Cycle by Second Generation Carbosilan Dendrimers

**DOI:** 10.1021/acs.biomac.2c00899

**Published:** 2022-11-29

**Authors:** Oscar Barrios, Belén G. Sánchez, Tamara Rodríguez-Prieto, Jesús Cano, Alicia Bort, Rafael Gómez, Inés Díaz-Laviada

**Affiliations:** † University of Alcalá, Department of Organic and Inorganic Chemistry, and Research Institute in Chemistry “Andrés M. Del Río” (IQAR), Madrid, 28871, Spain; ‡ Networking Research Center on Bioengineering, Biomaterials and Nanomedicine (CIBER-BBN), Madrid, 28029, Spain; § Ramón y Cajal Health Research Institute (IRYCIS), IRYCIS, Madrid, 28034, Spain; ∥ University of Alcalá, Biochemistry and Molecular Biology Unit. Department of Systems Biology and Research Institute in Chemistry “Andrés M. Del Río” (IQAR), Madrid, 28871, Spain; ⊥ Yale University School of Medicine, Vascular Biology and Therapeutics Program, New Haven, Connecticut 06520, United States

## Abstract

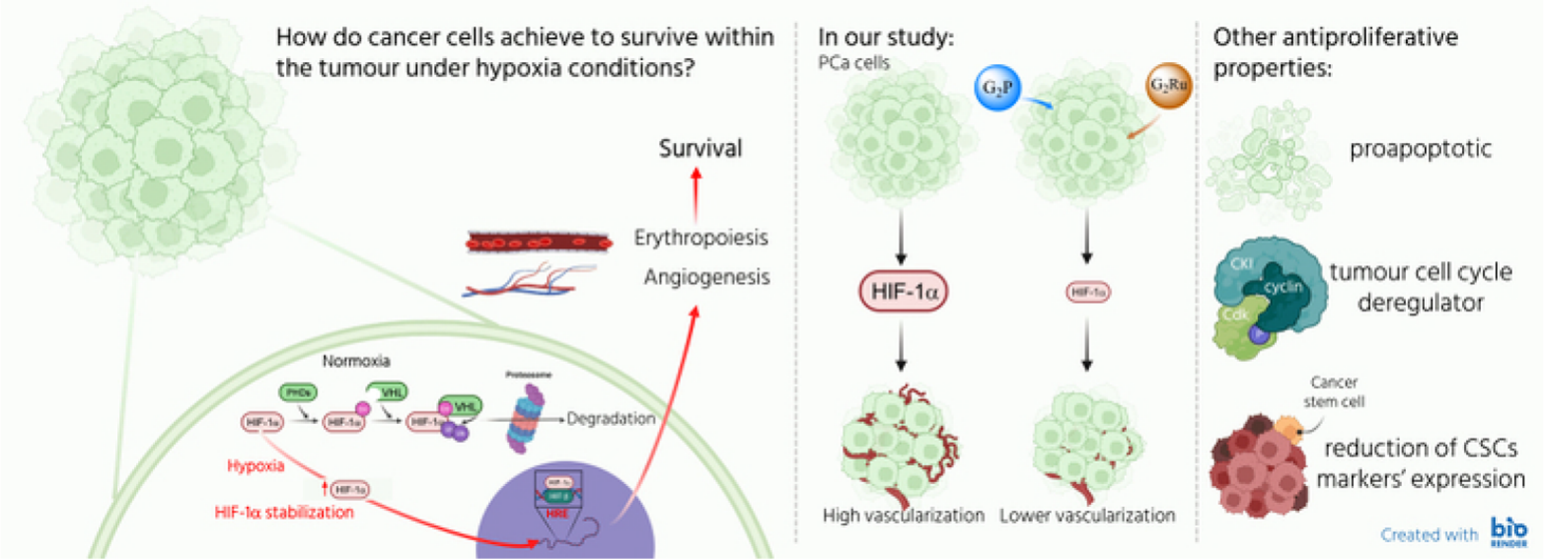

Current therapies against prostate cancer (PCa) disease, such as surgery, radiotherapy, or in last term chemical castration by androgen deprivation, have led to significant reduction of the incidence of PCa throughout the world. Worse prognosis is found in those patients which exhibit castration resistance, relapsing into the disease with even greater aggressiveness. Hypoxia cancer cell adaption has been observed to be closely connected to fatal prognostic tumor features. Therefore, hypoxia adaptive mechanisms of cancer cells have attracted large interest as a relevant biological target for treatment-resistant patients. Dendrimers have been established as a promising nanotechnological tool owing to their beneficial physicochemical features such as multivalency and monodispersity. Herein, we have completed a thorough study to better understand the effect within the cell of the already published ruthenium(II)-N-heterocyclic carbene metallodendrimer (G_2_Ru) that was able to drastically reduce HIF-1α stabilization and exhibited antiproliferative capability against androgen-sensitive (LNCaP) and androgen-resistant prostate cancer cells (LNFLU) *in vitro*. G_2_Ru, as well as its cationic imidazolium precursor (G_2_P), displayed scavenging properties against intracellular and externally stimulated ROS levels, which would presumably hinder the stabilization of HIF-1α by prolyl hydroxylase (PHD) inhibition. Furthermore, these dendrimers have shown considerably beneficial properties against tumor progression capability in terms of apoptosis, cell cycle, CSCs expression, and epithelial phenotype promotion. Taken all together, in this study we could demonstrate the extraordinary anticancer properties of NHC-based carbosilane dendrimers against androgen-resistant prostate cancer cells *in vitro*.

## 1 Introduction

Prostate cancer (PCa) was placed among the top five types of cancer with the highest incidence worldwide. In 2018, the number of incident cases exceeded one million people around the world, and deaths derived from PCa led to 389,989 people. Moreover, PCa incidence did not seem to have been reduced. Indeed, 1,017,712 of new cases are estimated up to 2040. Meanwhile, the number of deaths is expected to continue growing, reaching double the number of current prostate cancer derived deaths.^1^

**Figure 1:**
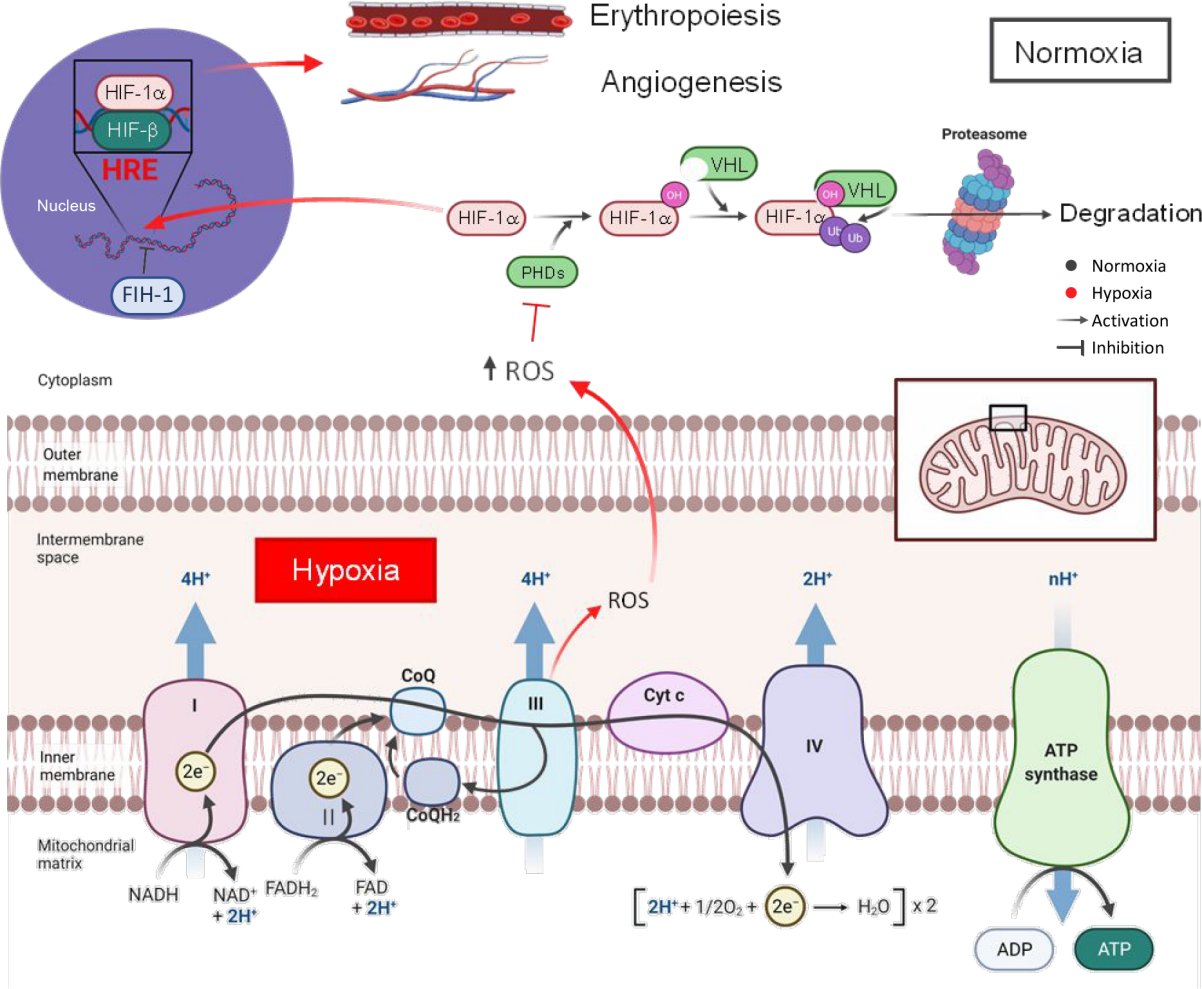
Mechanism of action and stabilization of HIF-1α under hypoxia. In normoxia, HIF-1α is hydroxylated within the cytoplasm by prolyl hydroxylases enzymes (PHDs). Thereby, Von Hippel Lindau protein (pVHL) recognizes previous hydroxylated residues and carry out the ubiquitin addition by its intrinsic ubiquitin ligase activity. Finally, proteasome degrades HIF-1α. Hypoxic HIF-1α stabilization is initiated by the ROS increase in the cytoplasm, provoked by the imbalance between pO_2_ and the electron transfer into the mitochondria. Then, PHDs action is inhibited and consequently, HIF-1α proteasome degradation does not take place, leading to HIF-1α translocation to the nucleus. Subsequently, HIF-1α, together with the HIF-β subunit, binds to the hypoxia response element (HRE) of the promoter regions, activating the transcription of angiogenic and erythropoietic genes, among others. Factor inhibiting HIF-1 act to impeding the HIF-1 complex binding to HRE element, thus hindering the promotion of angiogenic and erythropoietic genes. Scheme created with Biorender.com.

Current therapies against PCa differ essentially depending on the level of tumor extension. Clinically localized PCa is commonly treated with surgical castration or radiotherapy, but in the case of advanced-stage PCa, androgen-deprivation therapy (ADT) is the first line of treatment. However, despite frequently dramatic and sustained responses of many patients to ADT, castration-resistant prostate cancer (CRPC) occurs in 30% of patients. Patients relapse into the disease and tumor enlarges even with greater aggressiveness, displaying metastasis and drug resistance.^2^ Understanding the underlying mechanisms involved in the progression to CRPC will help to develop new therapeutic strategies to overcome aggressiveness and drug resistance. Several studies have shown that androgen withdrawal leads to a decrease in tumor oxygenation.^3^ Moreover, hypoxia can select androgen-independent prostate cancer cells, allowing the expansion of tumor cells with a more aggressive phenotype.^4^ Bhandari et al.^5^ observed that prostate tumors exposed to a low oxygen concentration presented a strong connection between the hypoxia microenvironment generated into the tumor and the decreased expression of several tumor suppressor genes. Therefore, hypoxia would be closely associated with the tumor progression, the treatment failure, and the fatal prognosis that recurrent castration-resistant PCa patients commonly present. Indeed, it has been observed that cancer cells can become resistant to hypoxia, and solid tumors survive in low oxygen conditions.^4^ This is of particular importance in the case of PCa, since the prostate gland is hypoxic compared to many other soft tissues, and hypoxia correlates with higher Gleason scores, resistance, and metastases.^5^ Cancer cells are capable of adapting to a low molecular oxygen concentration by activating transcription factors like the hypoxia-inducible factors (HIFs) involved in the adaptative response including the transcription of angiogenic and prosurvival genes as well as stem cell differentiation and proliferation.^6^ Under normal oxygen conditions, proline residues of HIF-1α subunit are hydroxylated by prolyl hydroxylase enzymes (PHDs), targeting HIF-1α for the subsequent ubiquitin addition by Von Hippel Lindau/E3 ligase protein (pVHL), initiating the proteasome degradation process (Figure 1). Nevertheless, during hypoxia, the post-translational stabilization of the HIF-α subunit in the cytoplasm maintains the factor activated promoting the expression of angiogenic genes. HIF-1α together with the HIF-β subunit mediates the response to hypoxia by the translocation to the nucleus where it binds to the hypoxia response element (HRE) of promoter regions, activating the transcription of hundreds of genes related with erythropoiesis, angiogenesis, cell proliferation, apoptosis, and survival, among others (Figure 1).^7,8^

The mechanism underlying HIF stabilization under hypoxic conditions is still unknown. However, according to other studies, hypoxia-induced reactive oxygen species (ROS) signals, formed by the imbalance between the pO_2_ and the electron flow into the mitochondria, may be implicated in the post-translational stabilization of HIF-1α by the inhibition of negative regulators such as prolyl-hydroxylases and the factor inhibiting HIF-1 (FIH-1) (Figure 1).^9−11^ In the tumor hypoxic environment, HIF-1α isoform modulates gene expression of proangiogenic factors such as VEGF (vascular endothelial growth factor),^12^ oncogenic growth factors like EGF (epidermal growth factor),^7^ mesenchymal regulators as N-Cadherin, and tumor invasion factors like TGF-β.^8^ In PCa, HIF-1 also promotes dysregulation of cancer-related genes implicated in the PI3K/Akt/mTOR signaling pathway and the differentiation of Cancer Stem Cells (CSCs) in PCa.^13^ Therefore, the importance of HIF-1 stabilization in the tumor progress makes HIF-1α a potential target in the research against cancer and, more specifically, against PCa.

In recent years, nanotechnology has been a participant in the great scientific advances carried out in the biomedical field.^14^ Among all the possibilities that it offers, dendritic polymer-based nanomedicines have recently acquired great relevance as smart and innovative nanomedicine systems against cancer. They have shown relevant cancer-cell targeting and internalization properties in cancer cell lines.^15,16^ Therefore, dendrimers have been positioned as potential structures for their use in biomedicine as anti-inflammatory, antiviral, anticancer agents, or even drug carriers. The dendrimer synthesis process offers very interesting physicochemical and biological characteristics as it is possible to control the nucleus, the extension of the branching units, and the number and nature of the terminal groups on the surface. Among the most outstanding properties of these structures, it is worth highlighting the monodispersity, which allows the control of their pharmacokinetics, and the multivalency, which allows potential cooperative effects and the design of dendrimers with different biological characteristics through their peripheral groups.^17^

**Figure 2:**
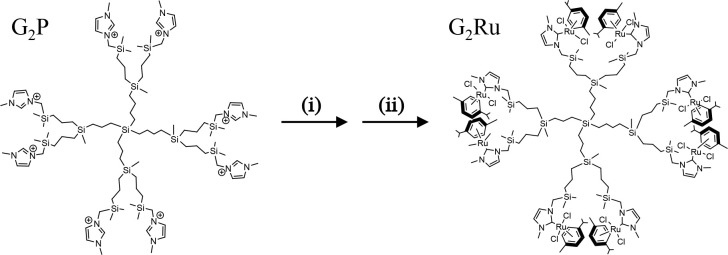
Synthesis of second generation metallodendrimer (G_2_Ru): (i) 4 AgO_2_, CH_2_Cl_2_, 8 h. (ii) 4 Ru[(*p*-cymene)Cl_2_]_2_.

The introduction of cisplatin as an anticancer agent, as well as the compounds derived from its composition, constitutes an advance in the design of new cancer therapies based on the use of metallopharmaceuticals.^18^ However, the new challenges in the current fight against cancer are based on counteracting the drug resistance, toxicity, or possible adverse effects that could affect healthy tissues. Recently, metallic compounds functionalized with N-heterocyclic carbene (NHC) ligands, commonly used for catalytic applications, have acquired a special interest in biomedicine due to the great variety of possibilities they offer, such as the control of their pharmacokinetics and reactivity, in addition to the easy access to them by organic synthesis.^19^*In vitro* and *in vivo* studies have shown the potential of Ru(II)-NHC complexes as optimal substitutes for cisplatin derivatives, as antitumor agents with toxicity values in healthy tissues much lower than those of cisplatin, elimination of resistance, or even alterations of metabolic processes essential for the survival of the tumor cells.^20−23^ Herein, a second generation carbosilane dendrimer functionalized with Ru(II)-NHC units (G_2_Ru)^24^ was studied as a possible anticancer agent in PCa, together with its precursor, based on the same carbosilane structure with imidazolium cationic groups on its periphery (G_2_P) (Figure 2).^25^ The aim of this work is centered on the combination of the inherent anticancer activity shown by the imidazolium or organometallic units with the multivalency supply by the dendritic structure.

In the present work we show that the compounds decrease ROS levels in prostate cancer cells. Hence, the *in vitro* study of the possible HIF-1α targeting effect of both dendrimers was analyzed. To ensure the most realistic results, two different PCa cell lines, differing on the sensitiveness to androgen deprivation, were utilized. G_2_Ru and G_2_P effects were studied on a sensitive prostate cancer cell line (LNCaP) and on an *in vitro*-generated resistant prostate cancer cell line (LNFLU) which exhibited resistance to Flutamide and Docetaxel, commonly used in clinic as prostate cancer chemotherapeutics.^26^ Thereby, in the current study, we expose the results of *in vitro* analysis related with the anticancer potential of these dendrimers and their HIF-1 targeting effect. The working hypothesis is focused on whether the reduction of ROS signals by these compounds may favor the degradation mechanism of HIF-1α counteracting the survival and development of PCa along with its biochemical consequences.

## 2 Materials and Methods

### 2.1 Chemicals

Second generation carbosilane metallodendrimer (G_2_Ru) and the corresponding precursor with imidazolium salt (G_2_P) were synthesized according to the protocol described below and reported elsewhere.^24,25^

### 2.2 Carbosilane Dendrimers

In this research, the dendrimers used to carry out the experiments, contained two different types of moieties. G_2_Ru bears in their structure Ru(II) N-heterocycle carbenes (Ru(II)-NHC) as functional groups, whereas precursor dendrimer (G_2_P) holds in their structure *N*-methyl imidazolium salts. The dendrimers were prepared in our research group to perform the experiments. In short, the procedure followed to obtain G_2_Ru consists of formation of Ag(I)-NHC units *in situ* using the precursor G_2_P and Ag_2_O, and subsequently carrying out a transmetalation reaction using [Ru(η^6^-p-cymene)Cl_2_]_2_. Dendritic systems were characterized by ^1^H and ^13^C NMR (Varian Unity VXR-300 NMR) at ambient temperature, elemental analyses (PerkinElmer 240C), and mass spectrometry (Agilent 6210 spectrometer (ESI) in the positive mode). Data are in agreement with those reported in the literature.^24,25^ Material characterization is available in the corresponding publications, cited before.

### 2.3 Cell Culture

The human prostate cancer cell line LNCaP was provided by the American Type Culture Collection (ATCC No. CRL-1740, Rockville, MD, USA). Cells were routinely cultured in RPMI-1640 medium supplemented with l-glutamine, 10% FBS, and 1% antibiotic purchased from Sigma-Aldrich (St. Louis, MO, USA). LNFLU, an androgen-independent cell line, was developed in the laboratory from LNCaP cells by adaptation to grow in the presence of the androgen receptor antagonist 2-hydroxyflutamide for two months.^26^

### 2.4 Intracellular ROS Quantification

LNCaP and LNFLU cells (3.0 × 10^5^ cells/cm^2^) were seeded in 6-well plates. After 48 h, cells were treated with G_2_Ru and G_2_P (3 and 10 μM) for 24 h. The levels of intracellular reactive oxygen species (ROS) were measured using the oxidation-sensitive fluorescent probe 2′,7′-dichlorofluorescin diacetate (DCF-DA, Sigma, St. Louis, MO, USA). Shortly, cells were harvested and incubated with 5 μM of DCF-DA for 30 min in the dark at room temperature. Finally, cells were digested with 0.25% trypsin and centrifuged at 1500 g for 5 min. Next, cells were incubated at 37 °C during 10 min in 500 mL of PBS, adding 4 μL of 50 mM *tert*-butyl hydroperoxide (Fisher Scientific, Foster City, CA, USA) into the positive control sample. Cells were centrifuged and resuspended in 500 μL of PBS 0.6 μg/mL propidium iodide (PI). The analysis was carried out using MacsQuant Analyzer 10 Flow Cytometer (Colonia, Germany), and the data was evaluated with MacsQuantifyTM Software (Miltenyi Biotech Inc., San Diego, CA, USA).

### 2.5 RT-qPCR

NZT Total RNA Isolation kit (Nzytech, Lisbon, Portugal) was used for RNA extraction. Then, using NZY First-Strand cDNA Synthesis Kit (Nzytech, Lisboa, Portugal), the corresponding cDNA was obtained from total RNA (2 μg). Subsequently, superoxide dismutase 2 (SOD2) and catalase (CAT) genes were quantified by qPCR (NZYSpeedy qPCR Green Master Mix (2×), ROX (Nzytech, Lisboa, Portugal) according to the manufacturer’s guide in a 10 μL final volume, using real-time PCR system 7500 (Applied Biosystems INC, Foster City, CA, USA). Gene-specific primers (SOD2-F 5′-GGCCTACGTGAACAACCTG A-3′, SOD2-R 5′-TTCTCCACCACCGTTAG GG-3′; CAT-F5′-GTGCGGAGATTCAACACTGCCA-3′; CAT-R 5′-CGGCAATGTTCTCACACAGACG-3′); Actin-F5′-AGAAGGATTCCTATGTGGGCG-3′; Actin-R5′-CATGTCGTCCCAGTTGGTGAC-3′ were used to amplify SOD2, CAT, and Actin (used as housekeeping gene).

### 2.6 Western Blot Analysis

Proteins were extracted by lysing cells at 4 °C in homogenization buffer (50 mM Tris-HCl pH 7.4; 41 mM Triton X100, NaVO_3_, 10 mM β-glycerophosphate, 5 mM NaF, 2 μg/mL leupeptin, 10 μg/mL aprotinin, 0.1 mM PMSF), followed by microcentrifugation. Twenty micrograms of total protein was separated by SDS-PAGE electrophoresis, and then, proteins were transfected to a PVDF membrane. After washing with TTBS, membranes were incubated overnight at 4 °C with the primary antibody. Next, membranes were incubated with peroxidase-labeled IgG anti-rabbit (Cell Signaling Technology, MA, USA) or IgG anti-mouse (Sigma, ST. Louis, MO, USA) secondary antibodies for 2 h at room temperature. The immune complex was developed by ECL system (Cell Signaling Technology, Danvers, MA, USA). Protein expression was measured using ImageJ software (National Institutes of Health, Bethesda, Maryland, USA), normalizing to the indicated housekeeping protein and expressed as fold changes respect to the control sample. β-Actin (Sigma, St. Louis, MO, USA), ALDH1-A1 (Cell Signaling Technology, MA, USA), CD133 (Cell Signaling Technology, MA, USA), VEGF (Abcam, MA, USA), HIF-1α (Abcam, MA, USA), cyclin D1 (Sigma, ST. Louis, MO, USA), p21 (Cell Signaling Technology, MA, USA), Akt (Cell Signaling Technology, MA, USA), p-Akt (Cell Signaling Technology, MA, USA), mTOR (Cell Signaling Technology, MA, USA), p-mTOR (Cell Signaling Technology, MA, USA), and E-Cadherin (Cell Signaling Technology, MA, USA) were used as primary antibodies.

### 2.8 Cell Viability

Cell viability was analyzed by MTT assay. Cells were harvested (1.5 × 10^5^ cells/well) into 12-well plates. After 48 h until full adhesion of cells, they were treated with 3 and 10 μM G_2_Ru and G_2_P for 24 h. After treatment, 100 mL of MTT 3-(4,5-dimethyl-2-thiazolyl)-2,5-diphenyl-2*H*-tetrazolium bromide) dye solution (Sigma-Aldrich) was added to each well and incubated at 37 °C for 1 h. Subsequently, medium was withdrawn, and formazan crystals were dissolved with 2-propanol. The, the optical density of each was measured by a microplate reader (iMARK, Bio-Rad Laboratories, Inc., Hercules, CA, USA) at 595 nm wavelength. Cell viability was calculated as the percentage of viable cells with respect to the vehicle-treated sample., which was assigned with 100% viability.

### 2.9 Flow Cytometry for Apoptosis

LNCaP and LNFLU cells (4.5 × 10^3^ cells/cm^2^) were grown in 6-well plates for 24 h until complete adhesion. After 24 h, cells were treated with 3 and 10 μM G_2_Ru and G_2_P for 24 h. Subsequently, apoptosis was evaluated at 24 h following treatment using an Annexin V-fluorescein isothiocyanate (FITC) Apoptosis Detection kit according to the manufacturer’s instructions (BD Biosciences, San Diego, CA USA). Briefly, cells were digested with 0.25% trypsin for 5 min. Cells were then centrifuged at 1500 g for 5 min and incubated in 0.5 mL of binding buffer (10 mM HEPES, pH 7.4, 150 mM NaCl, 2.5 mM CaCl_2_, 1 mM MgCl_2_, and 4% BSA), with 4 μg/mL Annexin V-FITC for 15 min. Cells were centrifuged and then resuspended in binding buffer with 0.6 μg/mL propidium iodide (PI). The analysis was carried out using MacsQuant Analyzer 10 Flow Cytometer (Colonia, Germany), and the data was evaluated with MacsQuantifyTM Software (Miltenyi Biotech Inc., San Diego, CA, USA).

### 2.10 Statistical Analysis

The obtained data were analyzed by GraphPad Prism 6.0 (La Jolla, CA, USA) and the results were expressed as the mean ± standard error. For multiple comparisons ANOVA and Tukey’s or Sidak’s test were performed. Statistically, significant differences were accepted for *p* < 0.05.

## 3 Results

### 3.1 Reduction of ROS by G_2_Ru and G_2_P Dendrimers

**Figure 3:**
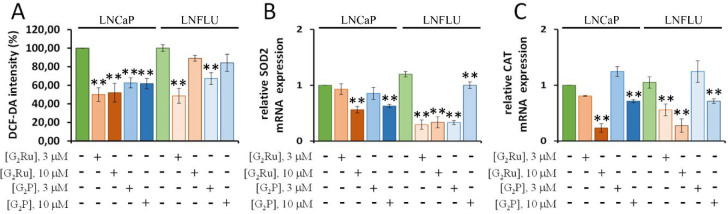
Treatment with the dendrimers significantly decreases the reactive oxygen species (ROS) levels in both sensitive (LNCap) and resistant (LNFLU) cells. A. ROS levels of LNCap and LNFLU when treated with G_2_Ru and G_2_P. Cells were treated with a 3 μM and 10 μM dose of dendrimer during 24 h. The histogram represents the fluorescence of DCFDA in treated cells with respect to nontreated cells. Data is represented as the percentage of fluorescence with respect to the control sample (*n* = 3). B. qPCR analysis of superoxide dismutase 2 (SOD2) mRNA levels. Cells were treated with 3 μM or 10 μM dose of dendrimer during 24 h. Data were normalized to vehicle-treated cells by the ΔΔ*C*_T_ method (*n* = 3). C. qPCR analysis of catalase (CAT) mRNA levels. Cells were treated with 3 μM or 10 μM dose of dendrimer during 24 h. Data were normalized to vehicle-treated cells by ΔΔ*C*_T_ method (*n* = 3). **, *p* < 0.01 significant differences between dendrimer-treated cells and the respective vehicle-treated cells by ANOVA test and Tukey test for multiple comparisons.

DCF-DA assay by flow cytometry was conducted for the elucidation of the effect of the dendrimers in ROS production. Two different doses of each dendrimer were used, 3 and 10 μM, to treat the prostate cancer cell lines LNCaP and LNFLU. The LNCaP cells depend on androgens for growing, while the LNFLU cell line was adapted to grow in the presence of the anti-androgen 2-hydroxiflutamide, thus acquiring an androgen-insensitive (or resistant) state. Flow cytometry histograms were analyzed for the acquirement of intracellular ROS levels (see Supporting Information, Figure S1). Figure 3A depicts the scavenging of free radicals by the metallodendrimer (G_2_Ru) and the precursor (G_2_P) dendrimer after 24 h treatment in LNCaP and LNFLU cells. The scavenging plot suggests that G_2_Ru reduced free radicals to about half of the intracellular ROS at both doses, while the precursor dendrimer only scavenged 40% in LNCaP cells compared to LNCaP vehicle-treated cells. Meanwhile, significantly reduced LNFLU intracellular ROS levels were only found when treating with a 3 μM dose of G_2_Ru and G_2_P, displaying 50% and 30% scavenged intracellular ROS, respectively, comparing to LNFLU vehicle-treated cells. Therefore, metallodendrimer held better scavenging capacities than its precursor, possibly due to the introduction of Ru(II)-*p*-cymene ligands all around its surface. Interestingly, both dendrimers do not show a dose–response effect on their ROS scavenging ability. It is necessary to consider that redox homeostasis of the cell represents a complex regulation system which not only depends on enzymatic and nonenzymatic mechanisms but also is regulated by the proliferation state and mitotic rate of the cell.^27,28^ Therefore, increasing the concentration of both dendrimers could mask possible higher changes in ROS levels due to parallel cellular changes affecting cell proliferation, such as cell cycle regulation and cell viability.

To clarify whether the ROS depletion was triggered by the reduction of the expression of ROS scavenging enzymes or nonetheless by an intrinsic scavenging feature of the dendrimers, qPCR experiments were carried out to determine the expression of superoxide dismutase 2 (SOD2) and catalase (CAT) genes (Figure 3B and C). In both cases, mRNA levels did not show any sufficient increase to explain the reduction of ROS levels within the cell. Meanwhile, mRNA levels of the two enzymes diminished when cells are treated with a dendrimer dose of 10 μM in both cell lines (Figure 3B and C).

In general, both dendrimers do not apparently show an explicit capacity to induce the transcription of ROS scavenging enzymes such as SOD2 or CAT, for the detoxification of intracellular ROS. Indeed, the decrease in the expression of SOD2 and CAT could be explained by an adaptive modulation of the gene expression to maintain the cell redox status, in response to the drop in the intracellular ROS levels triggered by a hypothetic intrinsic scavenging ability of the dendrimers.^27,29,30^ Herein, we only have studied the gene expression of two antioxidant enzymes implicated in the broad regulating network that adjust ROS intracellular levels. Hence, although some data in Figure 3 could entail some doubts about the dendrimer scavenging mode of action, a broader study would be necessary in order to clarify the mechanism of ROS scavenging elicited by the dendrimers. This work would involve studying the whole regulating-ROS mechanism and the effect on cell proliferation and other physiological processes triggered by these dendrimers on the intracellular ROS homeostasis.

Imidazolium derivatives have been demonstrated to be promising antioxidant systems effectively attenuating ROS in a dose-dependent manner. Their antioxidative properties were proposed to be through the direct neutralization of hydrogen peroxide or other radical species by carbene or bisimidazolidine and on the basis of the equilibrium between imidazolium, carbene, and bisimidazolidine species.^31^ Respecting the role of metal complexes, antioxidant and oxidant effects have been observed. As oxidant, they increase the level of ROS by targeting antioxidant enzyme systems or at the mitochondrial level.^32^ Regarding antioxidant properties, as a way to retard oxidative damage, the control of the redox potential of the metal center seems to be crucial.^33,34^ We show here that both the second-generation carbosilane metallodendrimer G_2_R and its corresponding precursor G_2_P exerted an antioxidant effect on androgen-sensitive as well as on androgen-resistant prostate cancer cells.

### 3.2 Scavenging Effect of G_2_Ru and G_2_P against Stimulated ROS Production

**Figure 4:**
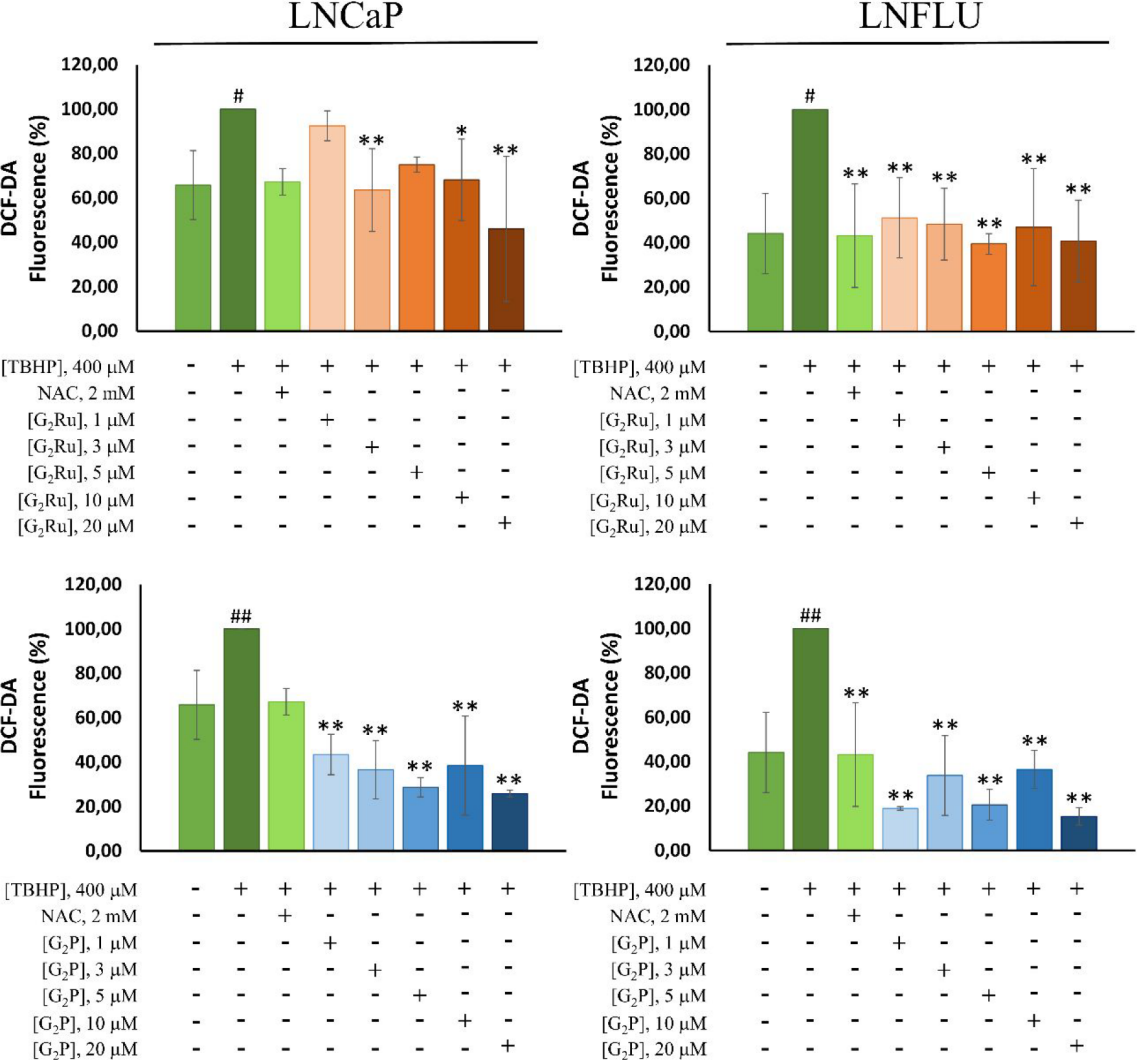
Treatment with G_2_Ru or G_2_P dendrimers prevents the reactive oxygen species (ROS) increase induced by an external stimulus in both sensitive (LNCaP) and resistant (LNFLU) cells. ROS levels in LNCaP and LNFLU cells when stimulated with TBHP and treated with G_2_Ru or G_2_P. Cells were cultured and treated with a 1, 3, 5, 10, and 20 μM dose of dendrimers during 10 min. NAC 2 mM was used as a positive control. The histogram represents the fluorescence of DCFDA in treated cells with respect to TBHP-treated. Data is represented as the percentage of fluorescence with respect to the TBHP-treated cells. (*n* = 3). ##, *p* < 0.01 significant differences between TBHP-treated and vehicle-treated cells. **, *p* < 0.01, *, *p* < 0.05 significant differences between dendrimer-treated and TBHP-treated cells by ANOVA test and Tukey test for multiple comparisons.

The dendritic complexes demonstrated reduction of intracellular basal levels of ROS, without increasing scavenging enzyme expression. Therefore, to demonstrate the scavenging capacity of these dendrimers, their antioxidant ability against exogenous ROS stimulation was assessed (Figure 4). Flow cytometry histograms were analyzed for the acquisition of ROS levels (see Supporting Information, Figure S2). Both cell lines were exposed for 10 min to 400 μM *tert*-butyl hydroperoxide (TBHP) treatment after being treated with either G_2_Ru or G_2_P during 10 min with a broad range of doses of 1, 3, 5, 10, and 20 μM. *N*-Acetylcysteine (NAC) 2 mM was used as a positive control, since it represents a well-established scavenger against intracellular ROS. It could be observed that 10 min of treatment with TBHP was enough to significantly increase the ROS production within the cells when compared to vehicle-treated cells in both cell lines (Figure 4). The two dendritic complexes significantly prevented the TBHP-induced rise of ROS production, reducing the ROS levels to similar values to those found for the vehicle-treated cells. Therefore, G_2_Ru and G_2_P seem to prevent the increase in ROS production even with only 10 min of treatment at a wide range of doses. Remarkably, although no dose–response effect linked to their scavenging ability could be found, the dendrimer scavenging capacity was completely demonstrated since they have been observed to be able to revert the stimulation of ROS production by TBHP to the vehicle-treated cell state, and the results are comparable to the effect found for the well-established scavenger, NAC.

### 3.3 The Dendritic Complexes Hinder the Adaptation to Hypoxia of the Androgen-Sensitive and Androgen-Resistant Cell Lines

**Figure 5:**
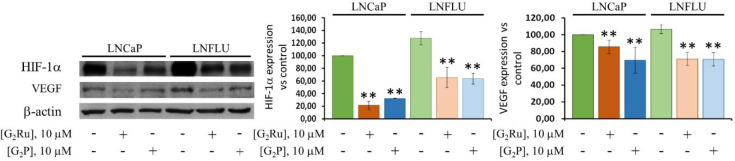
G_2_Ru and G_2_P reduce hypoxia-induced factor (HIF-1α) expression in both cell lines and trigger the consequent decrease of the vascular endothelial growth factor (VEGF) expression. Western blot analysis of HIF-1α and VEGF. Cells were cultured and treated with 10 μM of G_2_Ru or G_2_P dendrimers during 24 h. β-actin was used as a loading control. Data were normalized to vehicle-treated cells (*n* = 3). **, *p* < 0.01 significant differences between dendrimer-treated cells and the respective vehicle-treated cells by ANOVA test and Tukey test for multiple comparisons.

ROS play an important role in cellular homeostasis activating hypoxia-inducible signaling pathways and nuclear transcriptional responses that provide adaptation and increased stress resistance in cells. A central signaling molecule activated by hypoxia is the hypoxia-inducible factor HIF-1α which is a critical molecule connecting the cell redox status with nuclear gene expression. To determine whether the scavenging capacity of the dendrimers resulted in a modification of HIF-1α expression, Western blot analysis was carried out in prostate cancer cells treated with the dendrimers. Markedly, both cancer cell lines already exhibited a high level of HIF-1α, suggesting a nearly hypoxic condition within the cell, able to stabilize HIF-1α (Figure 5). Furthermore, HIF-1α expression was significantly higher in the androgen-insensitive cell line LNFLU, apparently confirming the close relationship between resistance and hypoxia adaptation. As shown in Figure 5, both dendritic systems significantly reduced HIF-1α expression in both sensitive and resistant cell lines. The Western blot densitometric analysis showed that the metallodendrimer triggered an 80% decline on the expression of HIF-1α in the LNCaP cell line with respect to the vehicle-treated cells, while the precursor provoked a reduction of about 70%. In the case of LNFLU cells, both treatments presented a similar trend, although they caused a 60% drop with respect to the vehicle-treated sample.

Since the vascular endothelial growth factor (VEGF) represents the main target of HIF-1α, we measured its expression in the prostate cancer cells treated with the dendrimers. According to the higher expression of HIF-1α, LNFLU cells showed increased levels of VEGF compared to LNCaP cells (Figure 5). A decrease in VEGF expression was found in cells treated with the dendrimers, which correlates with the reduction observed in HIF-1α levels and suggests a direct effect of the HIF-1α drop into the expression of VEGF. In the sensitive LNCaP cell line, G_2_Ru and G_2_P significantly reduced VEGF expression by 15% and 30%, respectively (Figure 5). The resistant cell line LNFLU suffered a decrease in VEGF expression of 30% with the dendrimers. Moreover, preliminary results show that both dendrimers were able to reduce VEGF expression even in the presence of cobalt chloride (II), a well-known inhibitor of HIF-1α proteasomal degradation (Supporting Information Figure S3), suggesting a potent effect of G_2_Ru and G_2_P.^35^ Therefore, the scavenging ability of both dendritic systems could be blocking the adaptation of these PCa cell lines to hypoxia, hindering HIF-1α stabilization by the ROS decrease and thus preventing VEGF expression. By this mechanism, dendrimers would be able to impact prostate cancer cells, scavenging the ROS increase produced by the aberrant and uncontrolled proliferation of cancer cells and reducing the vascularity of the surrounds of solid tumor masses since VEGF transcription is reduced.

### 3.4 The Dendrimers Inhibit Prostate Cell Proliferation and the PI3K/Akt Signaling Pathway

**Figure 6:**
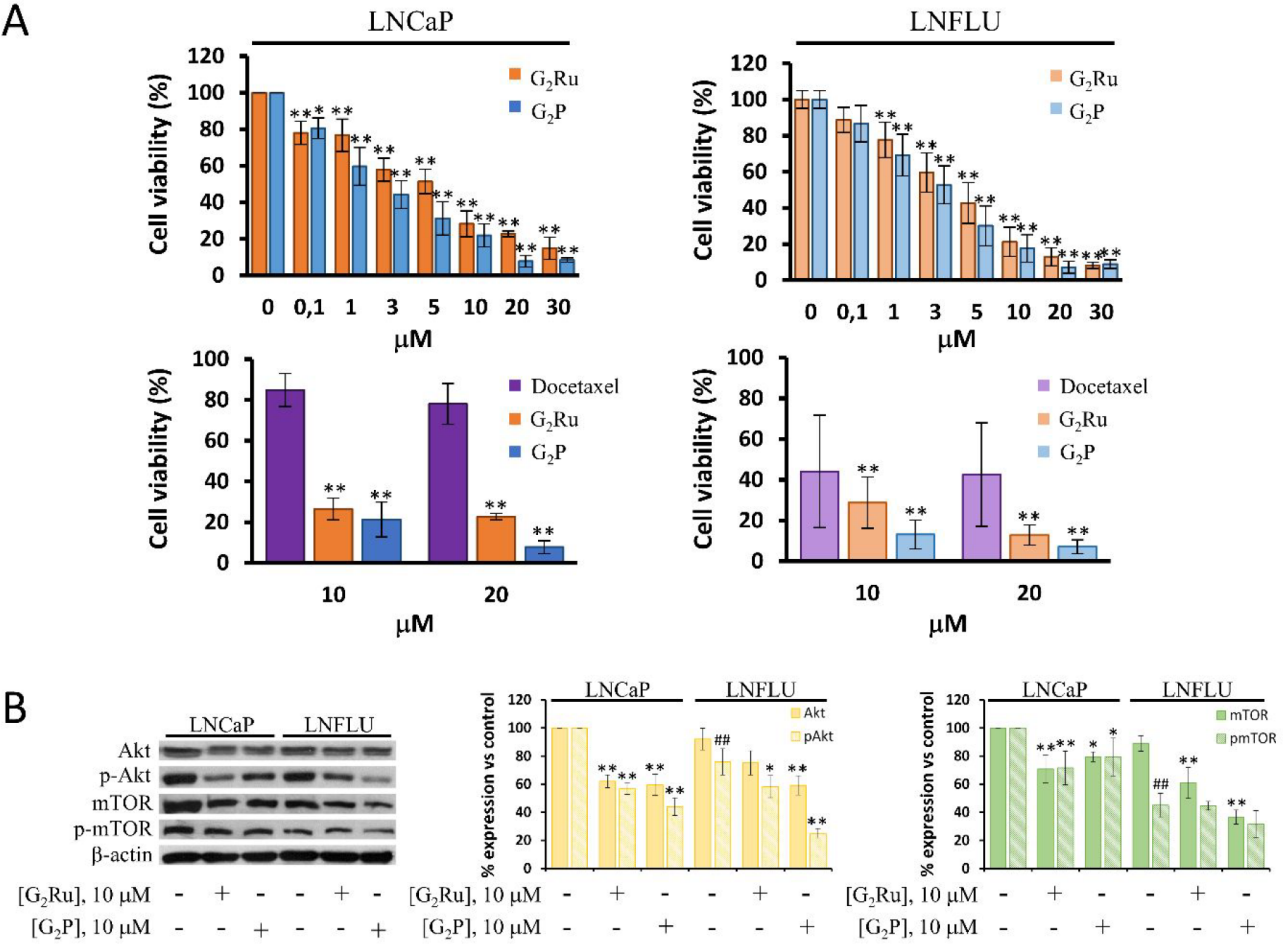
G_2_Ru and G_2_P inhibit cell growth in both sensitive (LNCaP) and resistant (LNFLU) prostate cancer cells. A. Cell viability of LNCaP and LNFLU cells treated with increasing doses of G_2_Ru or G_2_P dendrimers during 24 h assessment by MTT. G_2_Ru and G_2_P antiproliferative activity at 10 and 20 μM dose was compared with the antiproliferative capacity at the same doses of Docetaxel, a well-established chemotherapeutic agent in PCa. Data is represented as percentage of absorbance in dendrimer-treated cells with respect to vehicle-treated cells (*n* = 3). B. Western blot analysis of Akt, p-Akt, mTOR, and p-mTOR. Cells were cultured and treated with 10 μM of G_2_Ru or G_2_P dendrimers during 24 h. β-actin was used as a loading control. Data of densitometric analysis were normalized to vehicle-treated cells (*n* = 3). **, *p* < 0.01 and *, *p* < 0.05 significant differences between dendrimer-treated cells and their respective vehicle-treated or Docetaxel-treated cells; ##, *p* < 0.01 significant differences between vehicle-treated LNCaP cells and vehicle-treated LNFLU cells by ANOVA test and Tukey test for multiple comparisons.

To corroborate the adverse effect of the dendrimers on prostate cancer cell proliferation, we determined cell viability by MTT. As shown in Figure 6A, both dendrimers caused a decrease in the cell viability with IC50 values in the low micromolar range. A major potency in reducing the proliferation of LNCaP cells was found with the precursor dendrimer G_2_P, which showed an IC50 value of 1 μM compared to the metallodendrimer which displayed a IC50 value of 3 μM. Surprisingly, although it could be expected to find a lower activity of both dendrimers on the resistant cell line, G_2_Ru and G_2_P exhibited IC50 values of 3 μM, as in LNCaP cells, suggesting an androgen-resistant independent effect of both dendrimers on PCa cell lines. Furthermore, comparation with results of a drug control is necessary for dendrimer validation. Docetaxel represents a widely used chemotherapeutic agent and has been identified as a well-established drug control in PCa.^36^ Therefore, G_2_Ru and G_2_P antiproliferative capacity was compared to the Docetaxel antiproliferative ability at two concentrations, 10 and 20 μM. Both dendrimers showed a higher antiproliferative effect than Docetaxel in both LNCaP and LNFLU cells, and significant differences were found.

Additionally, the phosphatidyl inositol 3-kinase (PI3K)/Akt signaling pathway is the main prosurvival pathway implicated in prostate cancer progression and the development of castration resistance.^37^ Moreover, it has been demonstrated that the PI3K/Akt signaling pathway is involved in the resistance response to hypoxia and regulates the expression of HIF-1α.^38^ To further investigate the effect of dendrimers on prostate cancer cells, the PI3K/Akt signaling pathway was examined in this study. As shown in Figure 6B, basal levels of Akt were significantly reduced by the treatment with G_2_Ru and G_2_P dendrimers on both sensitive LNCaP and resistant LNFLU cell lines. Likewise, phosphorylated Akt levels were reduced similarly in dendrimer-treated cells. In addition, in mTOR, the main downstream substrate of Akt, basal levels were significantly reduced with the treatment with the dendrimers, even with a higher efficiency on the resistant cell line. Akt activates mTOR by phosphorylation of Ser-2448. To corroborate the inhibition of Akt induced by the dendrimers, we determined the phosphorylation of mTOR. As shown in Figure 6B, phosphorylated mTOR significantly declined in LNCaP cells treated with either G_2_Ru or G_2_P dendrimers through a similar trend. However, in LNFLU cells, although p-mTOR levels were markedly low in dendrimer-treated cells, no significance differences were obtained probably due to the low basal p-mTOR levels found in these cells.

### 3.5 Apoptosis Promotion on LNCaP and LNFLU Cell Lines by the Treatment with the Dendritic Systems

**Figure 7:**
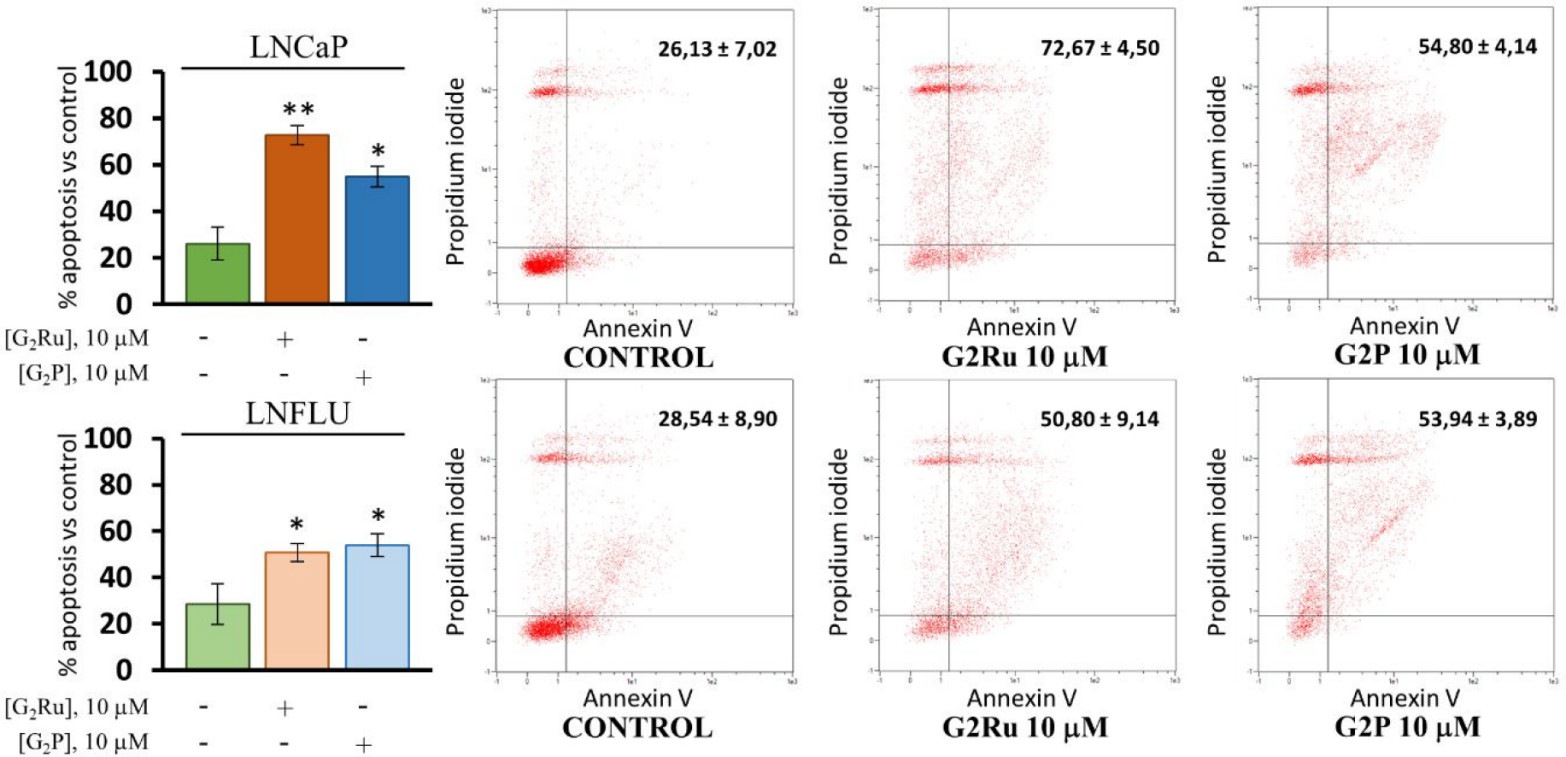
G_2_Ru and G_2_P promote apoptosis in both androgen-sensitive (LNCaP) and androgen-resistant (LNFLU) prostate cancer cells. Flow cytometry assay for the detection of apoptotic cells by Annexin V staining. Cells were cultured and treated with 10 μM G_2_Ru or 10 μM G_2_P dendrimers during 24 h. Ten thousand cells of each group were measured per experiment (*n* = 3). **, *p* < 0.01, *, *p* < 0.05 significant differences between dendrimer-treated cells and their respective vehicle-treated cells by ANOVA test and Tukey test for multiple comparisons.

PI3K/Akt activation is closely related with the induction of several cell survival mechanisms. Some of these mechanisms go from the suppression of proapoptotic factors to the activation of growth factors and prosurvival proteins triggering the singular aberrant proliferation ability of cancer cells.^39,40^ Thereby, to determine whether the metallodendrimer or its precursor dendrimer would be able to promote apoptosis in the prostate cancer cell lines, Annexin V and propidium iodide labeling of cells was conducted. Treatment of the androgen-sensitive LNCaP cell line with the dendrimers resulted in a significant increase in the percentage of late apoptotic cells (Figure 7). In the case of G_2_Ru, the percentage of late apoptotic cells approximately tripled, while G_2_P only doubled this percentage. On the other hand, the LNFLU androgen-resistant cell line showed smaller but significant changes with each dendrimer, reaching an approximately 25% increase. Therefore, G_2_Ru and G_2_P significantly promote programmed cell death in both prostate cancer cell lines.

### 3.6 G_2_Ru Markedly Interferes with the Regulation of Cell Cycle in LNCaP and LNFLU Cells

**Figure 8:**
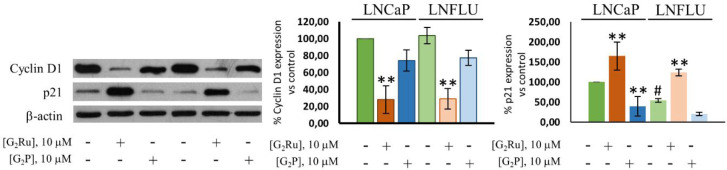
G_2_Ru and G_2_P vary the levels of the cell cycle regulators, p21 and cyclin D1. Cells were cultured and treated with 10 μM of G_2_Ru or G_2_P dendrimers during 24 h, and Western blot analysis of Cyclin D1 and p21 was performed. β-actin was used as a loading control. Data were normalized to vehicle-treated cells (*n* = 3). **, *p* < 0.01 significant differences between dendrimer-treated cells and their respective vehicle-treated cells; # *p* < 0.05 significant differences between vehicle-treated LNFLU cells and vehicle-treated LNCaP cells by ANOVA test and Tukey test for multiple comparisons.

It is widely known that the cell cycle arrest and apoptosis are closely connected, representing one of the main safety control strategies for preventing the cell from undergoing uncontrolled proliferation.^41,42^ Progression through the cell cycle has been demonstrated to be regulated by the activity of Cyclin-dependent kinases (Cdks). Cyclins represent the major regulator of Cdk activity since the formation of Cdk-cyclin complex is necessary for the activation of Cdks. Indeed, cyclins expression levels are modified during the cycle, thus controlling the progress through the different cell cycle phases. Additionally, Cdks can be suppressed by inhibitory regulatory proteins called Cdk inhibitor proteins (CKIs). The major exponent of CKIs is called p21, which forms a Cyclin-Cdk-CKI complex causing a rearrangement on the active site of Cdks. The DNA damage control system triggers the stimulation of p21 transcription by the activation of p53 gene regulatory protein. To further examine the mechanism underpinning the apoptotic effect induced by the dendrimers in prostate cancer cells, their effect on the expression levels of the cell cycle checkpoint regulators p21 and cyclin-D1 were studied by Western blot analysis. In contrast with the results described above, herein, G_2_Ru showed an intense effect on the expression of both regulatory proteins, while G_2_P hardly induced any effect on cyclin D1 and p21 expression levels (Figure 8). Notably, G_2_Ru triggered an intense decrease in cyclin D1 expression, with a similar potency in both cell lines, reducing the protein expression levels between 70% and 75%. By contrast, no significant changes were found for G_2_P. In the case of the Cdk inhibitor protein p21, it is worth mentioning that, as expected due to its greater cell proliferation capacity, lower basal p21 levels were found in the LNFLU cell line (Figure 8). Only G_2_Ru treatment triggers an upregulation on p21 expression. The treatment of cells with G_2_Ru provoked a 65–70% increase in p21 levels in both sensitive and resistant PCa cells. Meanwhile, G_2_P apparently increases p21 expression in both cell lines, although significant changes were only found in G_2_P-treated LNCaP cells. In conclusion, G_2_Ru apparently impedes Cdks activation by the downregulation of cyclin D1 and the upregulation of the cyclin inhibitor protein p21. Hence, taking into account the ineffective activity of G_2_P on the regulation of cyclin D1 and p21, the ruthenium(II) ligands’ introduction on the periphery of the dendrimer is clearly a determinant for impeding Cdks activation in both the androgen-sensitive and the androgen-resistant PCa cells.

### 3.7 Diminished Prostate Cancer Cell Line Malignancy with G_2_Ru and G_2_P Treatments

Malignant tumors not only present an aberrant and uncontrolled cell proliferation and growth process, but they are also capable of invading other tissues and generating a self-renewal source of cells within the tumor. Therefore, sensitizing cancer cells by reducing their malignancy represents a smart manner to eradicate cancer cells into a tissue. To migrate, epithelial cancer cells change their phenotype into mesenchymal cells, with a higher mobility and lesser adhesion, by the epithelial–mesenchymal transition (EMT). E-Cadherin, responsible for the formation of cell–cell adherent unions between epithelial cells and, thus, holding epithelial cells together, maintains the epithelial phenotype of the cell and impedes the EMT and the subsequent invasion of cancer cells to other tissues.^43,44^

**Figure 9:**
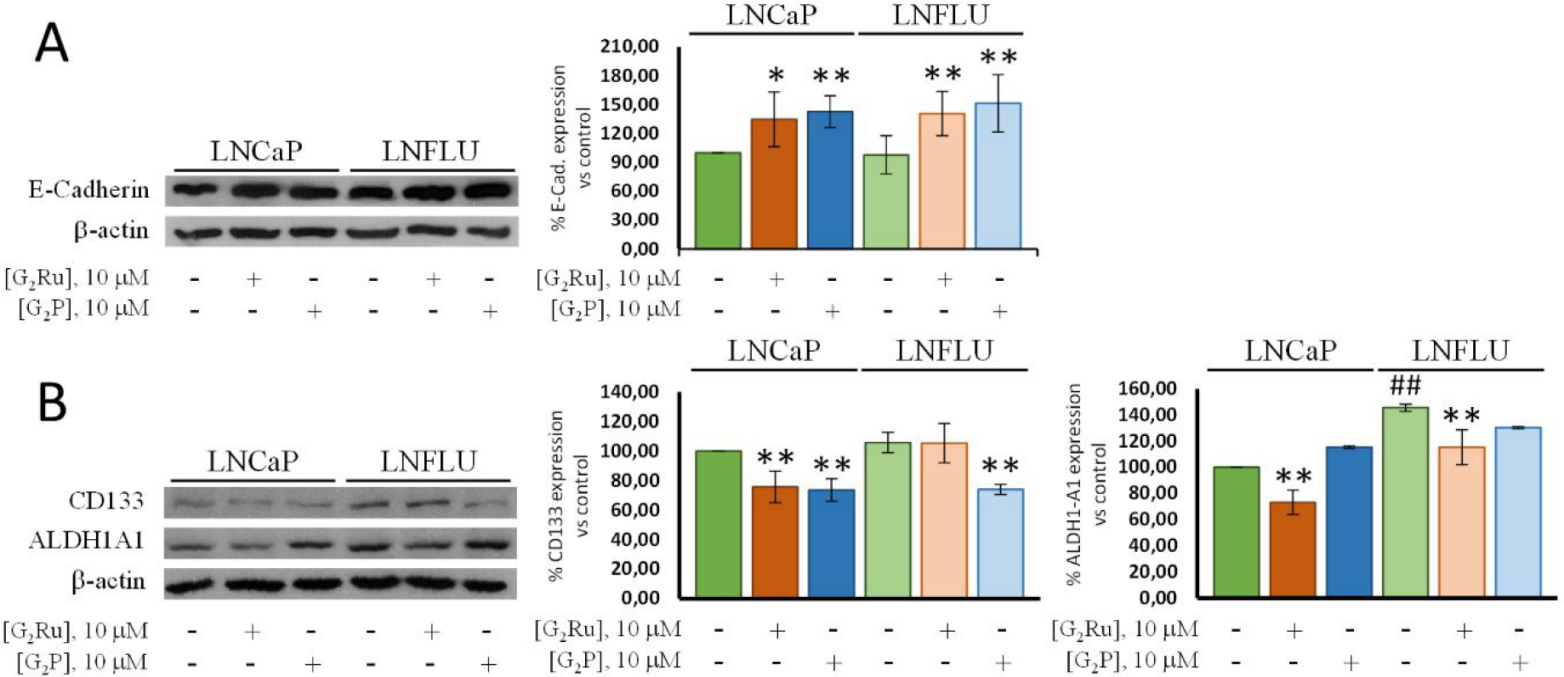
G_2_Ru and G_2_P reduce the malignancy of LNCaP and LNFLU cells by promoting an epithelial phenotype and reducing CSCs markers’ expression. A. Western blot analysis of E-Cadherin. Cells were cultured and treated with 10 μM of G_2_Ru or G_2_P dendrimers during 24 h. β-actin was used as a loading control. Data were normalized to vehicle-treated cells (*n* = 3). B. Western blot analysis of CD133 and ALDH1-A1. Cells were cultured and treated with 10 μM of G_2_Ru or G_2_P dendrimers during 24 h. β-actin was used as a loading control. Data were normalized to vehicle-treated cells (*n* = 3); **, *p* < 0.01, *, *p* < 0.05 significant differences between dendrimer-treated cells and the respective vehicle-treated cells; ##, *p* < 0.01 significant differences between vehicle-treated LNFLU cells and vehicle-treated LNCaP cells by ANOVA test and Tukey test for multiple comparisons.

To determine whether the dendritic complexes had an impact on EMT in prostate cancer cells, we determined the expression of E-Cadherine by Western blot. The dendritic systems were able to significantly increase the expression of E-Cadherin in the androgen-sensitive and androgen-resistant cell lines (Figure 9A). G_2_Ru and G_2_P showed a notable effect in the LNCaP cells, increasing the expression of E-Cadherin to 50% and 42%, respectively. In the LNFLU cell line the G_2_Ru and G_2_P dendrimers increased E-Cadherin expression to 37% and 30%, respectively. Hence, the dendritic systems significantly increase the expression of E-Cadherin in both cell lines. Although G_2_Ru seems to present a higher efficacy, both systems promoted an epithelial phenotype and presumably promote the cell–cell adherent unions, thus hindering the EMT.

Cancer stem cells (CSCs) represent an undifferentiated subpopulation of cells within the tumor, responsible for the initiation, maintenance, and dissemination of the tumor. These CSCs display features such as self-renewal, unlimited proliferation potential, and pluripotency. According to the CSC model, after the exposure to any anticancer therapy, CSCs would be the only subpopulation that survives and, therefore, due to their characteristics, are responsible for the tumor relapse. Cancer stem cell markers allow the study of CSCs within a cell line or even isolate CSCs by flow cytometry.^45,46^ One of the most studied biomarkers of CSCs is CD133, a transmembrane glycoprotein implicated in numerous molecular mechanisms of CSCs such as self-renewal and therapeutic resistance.^47,48^ On the other hand, the aldehyde dehydrogenases enzymes (ALDHs), responsible for the detoxification of exogenous aldehydes to their corresponding weak carboxylic acids, have also been utilized for the study of CSCs in cancer research.^45,49−51^ To investigate the effect of the dendrimer complexes on the prostate cancer stem cell differentiation, we determined the levels of CD133 and ALDH1A1 by Western bolt in the prostate cancer cell lines. As shown in Figure 9B, the androgen-resistant LNFLU cell line displayed higher levels of both CD133 and ALDH1A1 compared to their parental LNCaP cells, suggesting that resistance to androgens is accompanied by a reprogramming of cells into CSCs, as previously described.^26^ Herein, G_2_Ru and G_2_P treatment reduced CD133 expression to 24% and 26%, respectively, in the LNCaP cell line (Figure 9B). However, only G_2_P promoted significant changes in LNFLU cells, diminishing the expression of CD133 in a similar manner in LNCaP. Interestingly, the metallodendrimer G_2_Ru significantly reduced to 25% the expression of ALDH1A1 in both LNCaP and LNFLU cells. Not only do these results indicate a decrease in cancer stem cell markers’ expression, but these dendritic systems also clearly interfere with one of the main intracellular mechanisms of drug resistance. Therefore, both dendritic complexes have demonstrated the ability to reduce malignancy features of androgen-sensitive and androgen-insensitive prostate cancer cell lines by increasing a protein involved in the adherent unions between epithelial cells and reducing the typical markers of CSCs.

## 4 Discussion

In 1941, Huggins and Hodges^52^ observed that androgen deprivation therapy, led to tumor regression in advanced PCa patients. Since then, androgen deprivation therapy has been widely used to treat prostatic carcinoma. However, castration-resistant PCa (CRPC) usually relapse as primary tumor or dramatically as metastatic CRPC and the following line of treatment–driven by docetaxel– is hampered by resistance appearance. CRPC patients relapse into the disease with even worse prognosis due to, among other issues, a greater proliferation of cancer cells and a better adaptation to the hazard conditions found into the tumor. Therefore, new therapies have been studied in recent years to overcome drug resistance in CRPC. Herein, we have assessed the antitumoral properties of second-generation carbosilane dendrimers in two prostate cancer cell lines which differ in their sensitivity to androgens.

Furthermore, in this study we have also evaluated the possible beneficial effect of the introduction of eight ruthenium(II) ligands (G_2_Ru) by the formation of N-heterocyclic carbenes on the periphery of the precursor dendrimer containing imidazolium-terminated units (G_2_P), on the dendrimer anticancer properties.

In this study, we demonstrated that dendrimer complexes induce apoptosis and impact pro-survival signaling pathways in androgen-sensitive as well as androgen-resistant prostate cells. Moreover, dendrimer complexes decreased the expression levels of E-cadherin, CD133, and ALDH1A1, hallmarks of cancer aggressiveness and malignancy. Interestingly, the introduction of eight ruthenium(II) ligands notably improved the effect on cell cycle checkpoint proteins like Cyclin D1 and p21. Although the mechanism whereby dendrimers exerted their actions has not been elucidated, we show that they modified hypoxia adaptation mechanisms, since they decreased intracellular ROS concentration, HIF-1α levels, and VEGF expression, all of them key pathways involved in the response to hypoxia.

Our dendritic systems targeted HIF-1α by drastically reducing its expression, not only in the androgen-sensitive PCa cell line (LNCaP) but also in the androgen-resistant LNFLU cells. HIF-1α was observed to increase its expression by the increase in the intracellular ROS levels and, thus, inhibiting HIF-1α negative regulators that stimulate its degradation. Herein, G_2_Ru and G_2_P displayed scavenging capacities that reduced the intrinsic ROS levels of both cell lines. Furthermore, mimicking a possible hypoxia situation, endogenous ROS production was induced by the addition of TBHP, and both dendrimers showed an interesting ability to prevent ROS increase. Therefore, both dendritic systems would be able to prevent ROS increase into the cell, thus impeding the inhibition of the HIF-1α proteasome degradation process, stimulating its degradation and, consequently, reducing the cell adaptability to the tumorigenic hazard conditions.

HIF-1α activity was significantly reduced by G_2_Ru and G_2_P dendritic systems, which allow a significant decrease on VEGF expression, a specific HIF-1α regulated gene. Therefore, the HIF-1α targeting capacity of both dendrimers would trigger the reduction of vascularity in the area surrounding the tumor by the inhibition of angiogenesis induction. Although it was previously shown that androgens may regulate VEGF levels through the activation of HIF in androgen-sensitive tumors,^53^ we found that the androgen-resistant LNFLU cell line displayed higher levels of HIF-1α pointing to a hypoxia-resistant androgen-independent adaptation mechanism. It is worth noting that the dendrimers were able to decrease HIF-1α levels both in the androgen-dependent cell line and in the androgen-resistant cell line, suggesting that the effect induced by dendrimers is independent of androgen receptor.

Furthermore, MTT assay revealed the antiproliferative capacity of these dendrimers. It is worth mentioning that the IC50 values were significantly lower than those found with commonly PCa chemotherapeutic agents such as Flutamide or Docetaxel.^26^ In addition, both dendrimers inhibited the PI3K/Akt/mTOR signaling pathway, a survival mechanism essential to the normal cellular function and commonly source of function mutated in cancer models. This pathway is intimately connected with cellular processes such as apoptosis or cell cycle. Interestingly, loss of function mutations in tumor suppressors genes such as PTEN or p53 have been observed to induce HIF-1α stabilization.^8^ Moreover, HIF-1α hypoxia-independent stabilization by the PI3K/Akt/mTOR signaling pathway has been recently reported.^54,55^ Hudson et al.^56^ demonstrated the regulatory effect of the mammalian target of rapamycin (mTOR) on HIF-1α normoxic and hypoxic stabilization in PC-3 cells. Our observation of the inhibitory effects on the PI3K/Akt/mTOR signaling pathway exerted by the dendritic systems in LNCaP and LNFLU cells suggested that. in addition of the ROS depletion, the downregulation of the PI3K/Akt/mTOR activity could also be involved in the HIF-1α stabilization induced by dendrimers.

Cell proliferation is controlled by mitogenic pathways that lead to the expression of Cyclin D1 which in turn regulates cell cycle progression, as well as negatively by cell cycle arrestors like p53 or p21 proteins. Herein, G_2_Ru and G_2_P were seen to significantly induce apoptosis on both cell lines, overcoming PI3K/Akt/mTOR aberrant activity in LNCaP and LNFLU cells. Furthermore, it is worth mentioning that G_2_Ru drastically reduced cyclin D1 expression while markedly increasing expression of the Cdk inhibitor protein p21, that promotes cell cycle arrest. Interestingly, these findings suggest that G_2_Ru hinders the activity of the cyclin D1-Cdk complex on the progression of the cell cycle, not only by reducing the levels of cyclin D1 but also by blocking Cdk-cyclin complex activity by promoting p21 expression. Hence, G_2_Ru was observed to exhibit an explicit capacity to interfere with the aberrant cancerous cell cycle by reducing Cdk-cyclin complex activity and promoting apoptosis.

Finally, we found that both dendrimers increased E-Cadherin levels, apparently promoting an epithelial phenotype in both androgen sensitive and resistant PCa cells, presumably hindering epithelial-mesenchymal transition and thus reducing cancer cells’ invasion capacity. Furthermore, we observed that G_2_Ru and G_2_P significantly reduced CD133 and ALDH1A1, respectively. This phenomenon of cancer stem cell markers reducing capacity of both dendrimers could be of special interest in anticancer therapy, since they could reduce drug resistance and invasion characteristics of resistant PCa cells.

## 5 Conclusion

We have developed a smart and sophisticated alternative for prostate cancer therapy that showed higher potency than commonly chemotherapeutic agents. We could demonstrate the scavenging ability of these dendrimers in the cell lines studied, probably triggering the reduction of HIF-1α stabilization after G_2_Ru and G_2_P treatment in normoxia. In addition, the dendrimers were additionally able to prevent ROS increase, which would hypothetically inhibit HIF-1α stabilization in the near-hypoxic conditions. Considering the close connection between HIF-1α stabilization and proliferative cancer cell characteristics, these results are of great importance since they provide a tool to fight against adaptive survival in cancer cells, as well as in angiogenesis and metastasis. These dendrimers were observed to display considerable antiproliferative features, increasing apoptosis levels and, in the case of G_2_Ru, inhibiting cell cycle progression by interfering with cell cycle regulation. Furthermore, stem cell markers in both cancer cell lines were decreased by each dendrimer and prevented the epithelial–mesenchymal transition by promoting E-Cadherin expression and consequently cell–cell adherent unions. Remarkably, these systems have displayed a beneficial effect on androgen-resistant PCa cells, introducing themselves as a promising alternative tool in CRPC patients who do not respond to the current treatment against CRPC.

## Supporting Information Available

Fluorescence histograms referring to Figure 3 and Figure 4; Western blot of VEGF in cells treated with G_2_Ru and G_2_P and a positive control, CoCl_2_; Abbreviation list (PDF)
