# Supplementary material for: Alteration of the HIF-1α/VEGF Signaling Pathway and Disruption of the Cell Cycle by Second Generation Carbosilan Dendrimers
Source: Biomacromolecules. 2022 Nov 29;23(12):5043–55. doi: 10.1021/acs.biomac.2c00899 (PMC9748942; doi:10.1021/acs.biomac.2c00899)
Supplement: Supplementary file 1 — bm2c00899_si_001.pdf [file bm2c00899_si_001.pdf]

Alteration of the HIF-1α/VEGF Signalling Pathway and Disruption of the Cell Cycle by Second Generation Carbosilan Dendrimers

Oscar Barrios[1], Belén G. Sánchez[4], Tamara Rodríguez-Prieto[1,2,3], Jesús Cano[1,2,3], Alicia Bort[4,5], Rafael Gómez[1,2,3]*, Inés Díaz-Laviada[4]*

[1] University of Alcalá, Department of Organic and Inorganic Chemistry, and Research Institute in Chemistry "Andrés M. Del Río" (IQAR), Madrid, Spain.
[2] Networking Research Center on Bioengineering, Biomaterials and Nanomedicine (CIBER-BBN), Madrid, Spain.
[3] Ramón y Cajal Health Research Institute (IRYCIS), IRYCIS, Madrid, 28034, Spain.
[4] University of Alcalá, Biochemistry and Molecular Biology Unit. Department of Systems Biology and Research Institute in Chemistry "Andrés M. Del Río" (IQAR), Madrid, Spain.
[5] Yale University School of Medicine, Vascular Biology and Therapeutics Program, New Haven, CT 06520

Supporting Information

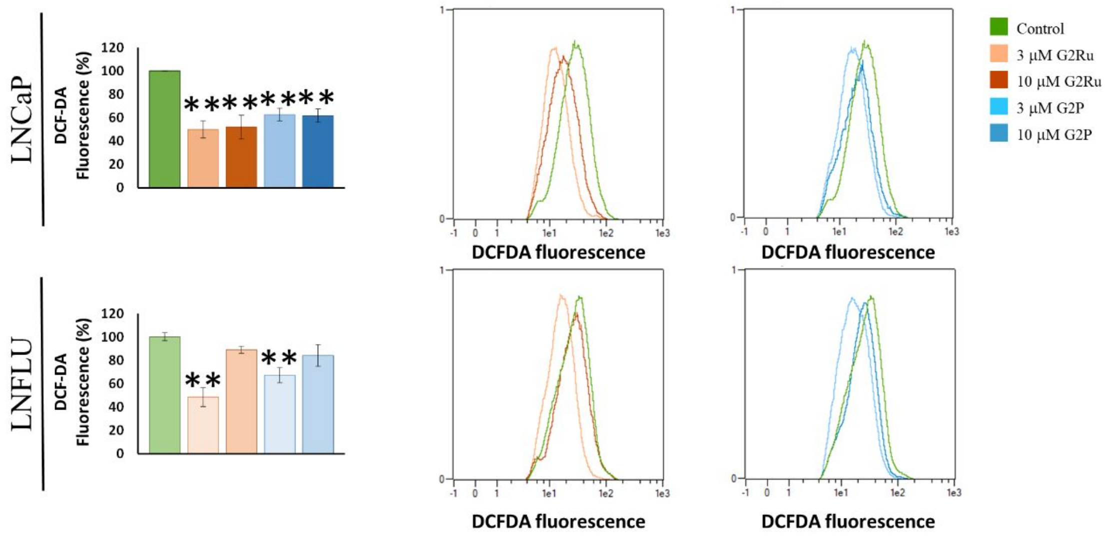

Figure S1. Representative overlay of the flow cytometry histograms for the elucidation of intracellular ROS levels.

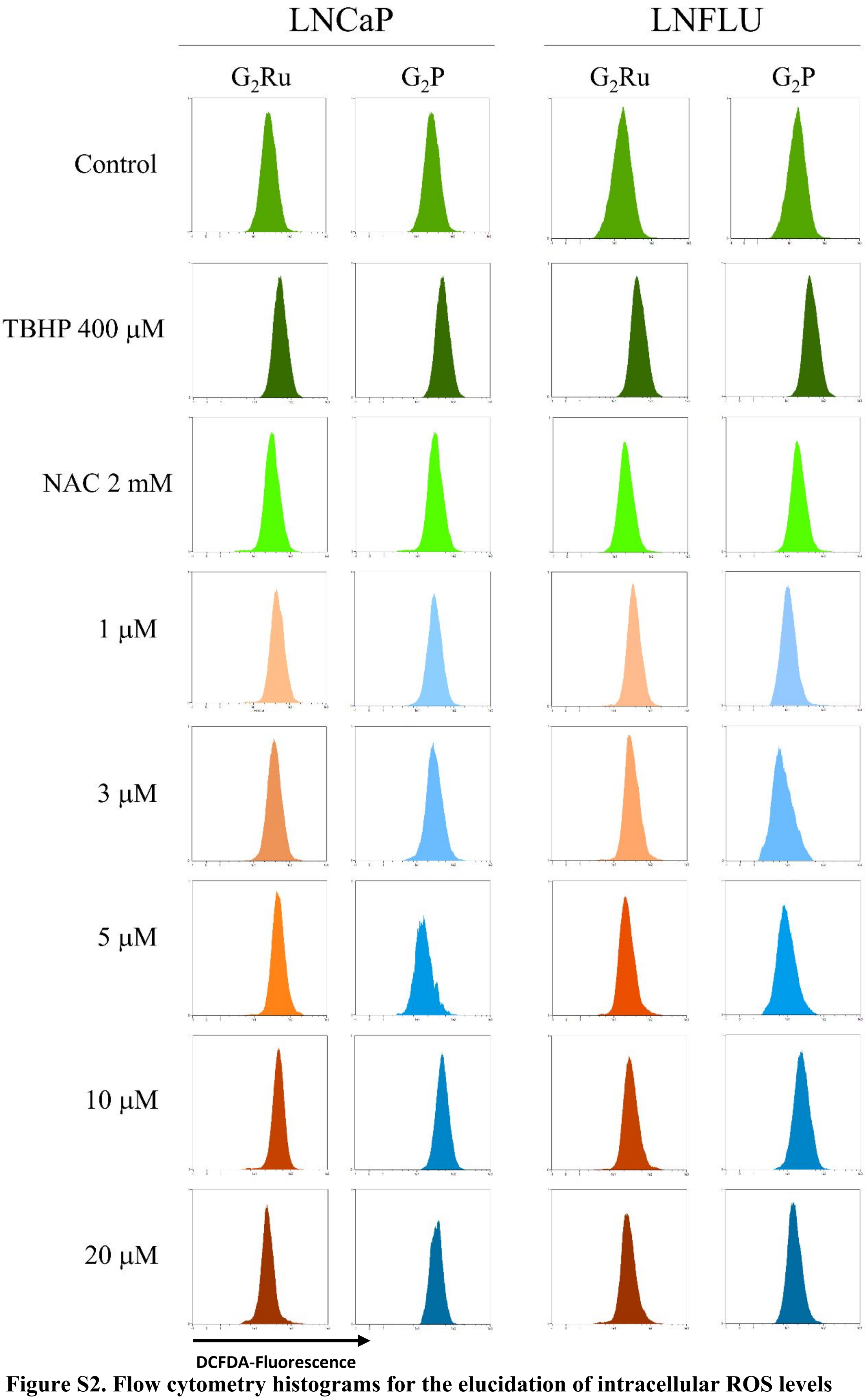

Figure S2. Flow cytometry histograms for the elucidation of intracellular ROS levels after TBHP stimulation.

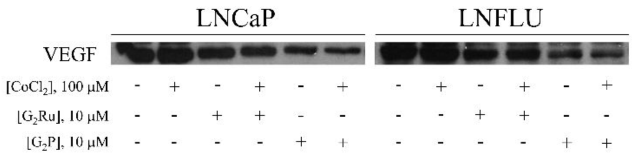

Figure S3. Both G2Ru and G2P dendrimers reduce VEGF expression in the presence of a HIF-1α proteosomal degradation inhibitor such as $CoCl_2$. Western-Blot analysis of VEGF. Cells were cultured and treated with 10 μM of G_2Ru or G_2P dendrimers. After 24 h, CoCl2-positive cells were treated with $CoCl_2$ 100 μM for 4 h.

LIST OF ABBREVIATIONS

ADT – Androgen-Deprivation Therapy

ALDH – Aldehyde Dehydrogenase

CAT – Catalase

Cdk – Cyclin-Dependent Kinases

CKI – Cdk Inhibitor Protein

CSC – Cancer Stem Cellv

CRPC – Castration-Resistant Prostate Cancer

DCFDA - 2',7'–dichlorofluorescein diacetate

EGF – Epidermal Growth Factor

EMT – Epithelial-Mesenchymal Transition

FIH-1 - Factor Inhibiting HIF-1

G_2P- Imidazolium cationic second generation carbosilane dendrimer

G_2Ru - ruthenium(II)-NHC derived second generation carbosilane dendrimer

HIF-1α - Hypoxia Inducible Factor- 1α

HRE- Hypoxia Response Element

LNCaP – androgen-sensitive prostate adenocarcinoma cell line

LNFLU – antiandrogen therapy-resistant cell line from LNCaP

mTOR – mechanistic Target of Rapamycin

NHC – N-heterocyclic carbene

PCa – Prostate cancer

PHD- Prolyl Hydroxylase enzyme

PI3K – Phosphatidylinositol 3-Kinase

pVHL- Von Hippel Lindau/E3Ligase protein

qPCR – quantitative Polymerase Chain Reaction

ROS – Reactive Oxygen Species

SOD2 – Superoxide Dismutase-2

TBHP – Tert-butyl Hydroperoxide

TGF-β - Transforming Growth Factor-1β

VEGF – Vascular Endothelial Growth Factor
